# Supplementary material for: Bicarbonate concentration as a predictor of prognosis in moderately severe COVID-19 patients: A multicenter retrospective study
Source: PLoS One. 2022 Jun 24;17(6):e0270141. doi: 10.1371/journal.pone.0270141 (PMC9232226; doi:10.1371/journal.pone.0270141)
Supplement: Attachment — Submitted filename: Response to Reviewers_20220406.docx [file pone.0270141.s001.docx]

**Reviewer 1**

The authors investigated the association between serum bicarbonate levels and clinical outcomes in patients with COVID-19.

Not only respiratory failure but also multiple organ failure causes the disorder of acid-base balance. Therefore, the interpretation of the disorder caused by COVID-19 is of great interest.

**Reviewer comment**

1) First, the author did not show the rationale for the definitions of high, normal, and low bicarbonate. As this study was conducted retrospectively, the standards should be shown in justification.

**Response**

Thank you for this suggestion. The clinical normal range for serum bicarbonate concentration varies by reference source[1]. Some literature has suggested the bicarbonate range of 21 to 27mEq/L as the normal range[2-5], and others have been suggested those of 21 to 28mEq/L[6]. We have divided bicarbonate into three groups based on previous literature (bicarbonate range of 21 to 27mEq/L).

We had revised as below (Methods L113);

The patients were classified into three categories according to the bicarbonate concentration based on previous literature: high (>27 mEq/L), normal (21 to 27 mEq/L), and low (<21 mEq/L) [12-15].

**Reviewer comment**

2) Second, the authors conducted multivariate Cox regression analyses with several models. However, the numbers of patients who reached the outcomes were around ten in each group.

Consequently, I think that the authors adjusted too many parameters. Due to the small number of patients, I do not think that appropriate multivariable Cox models were established in this study.

**Response**

We appreciate the opportunity to clarify this point. In Cox regression analysis, events per variable (EPV) of 10 is widely used as the lower limit for developing models. However, some studies have expressed concerns that the EPV ≥10 rule-of-thumb is not based on convincing scientific reasoning [7]. A recent study suggests that the EPV ≥10 criterion may be too strict [8, 9].

We believe that EPV < 10 is acceptable if the regression parameters can be estimated stably. We used six covariates (bicarbonate (categories), age, sex, treatment limitation, and SOFA respiratory) for the 25 outcomes (4.2 EPV). The standard error of the obtained estimates is small, indicating that the estimation is stable even with a small sample size.

| Term | OR | Std.error | P.value |
| --- | --- | --- | --- |
| High bicarbonate | 3.272 | 0.581 | 0.041 |
| Low bicarbonate | 4.022 | 0.506 | 0.006 |
| Age | 1.017 | 0.022 | 0.464 |
| Sex | 0.971 | 0.505 | 0.954 |
| Treatment limitation | 0.520 | 0.519 | 0.208 |
| SOFA respiratory | 1.685 | 0.468 | 0.265 |

Table. Detaille of primary analysis

However, as reviewers have commented, the small sample size is a valid concern, so we have added a limitation as below (Discussion L278);

The third was the use of a Cox model adjusted for six covariates for fewer outcomes. However, the standard errors of the regression coefficients were small, and we believe that we have achieved a stable estimation.

**Reviewer comment**

Third, the authors should explain “treatment limitation” in detail. There is no information in this manuscript.

**Response**

Thank you for this comment. As shown in L97-98, we had described “treatment limitation”.

We have added a more detailed explanation of “treatment limitation”.

L97,8 Methods;

Treatment limitations (defined as decisions to withdraw or withhold life-sustaining therapies such as mechanical ventilation, renal replacement therapy, extracorporeal membranous oxygenation, and cardiopulmonary resuscitation through Do Not Resuscitate (DNR) orders) [10].

**Reviewer comment**

Fourth, as the patients were not intubated, the authors should show how they calculated FiO2.

**Response**

Thank you for pointing this out. FiO2 for non-intubated patients was estimated based on previous literature. This estimation was used in the EPIC III study [11]. Estimation FiO2 table is available in CRF of EPIC III study (URL; https://www2.intensive.org/epic3/Home.asp)

We have added how to measure FiO_2_ in the Methods below;

FiO2 (estimated from devices (nasal cannula, nasopharyngeal catheter, face mask, face mask with reservoir) and oxygen flow [11].

**Reviewer 2**

The authors have investigated the utility of measuring HCO3 concentrations to predict prognosis in COVID-19 patients. The manuscript contains interesting findings that may be of interest to readers. However, the points discussed below need to be addressed.

**Reviewer comment**

1) The COVID-19 patients in this study had a higher pH on Day 1. Consider whether this should be attributed to disease specificity, and describe the differences in the pH results between non-COVID-19-infected and COVID-19-infected patients.

**Response:**

Unfortunately, we did not have data on non-COVID-19 infected patients. In the discussion section, we have acknowledged this issue as below;

Discussion L269;

Our patient had a slightly higher pH on Day 1 (Figure 2a), but whether this was due to disease specificity is not known because it was not compared to non-COVID-19 patients in our study. Although evidence is limited, higher pH has also been reported in patients with H1N1 influenza [12, 13]. Even before hypotension occurs, fever and sepsis can lead to an increase in pH via hyperventilation [14]. Therefore, the high pH on Day 1 may not be attributed to the disease specificity of COVID-19.

**Reviewer comment**

2) Table 2 shows that patients with COVID-19 have higher pH from Day 1 and that respiratory alkalosis is suspected due to the low PaCO2 values. The authors should evaluate the patients for alkalosis on Day 1.

**Response:**

Thank you for this insightful comment. We have added an evaluation of alkalosis on day 1. We defined metabolic alkalosis as pH >7.42 and bicarbonate >26 mmol per liter.

Respiratory alkalosis was defined as pH >7.42 and PaCO_2_ <38 mm Hg[15].

As a result, 15 had respiratory alkalosis, 1 had metabolic alkalosis, and 1 had both on Day 1.

We have revised as below (Results L179) ;

On day 1, 15 had respiratory alkalosis, 1 had metabolic alkalosis, and 1 had both.

**Reviewer comment**

3) In the discussion, the authors describe metabolic alkalosis as an important change in severe disease (lines 246–249). According to the graph of pH changes shown in Table 2, the pH decreased on Day 4 in the high HCO3 group. An abnormal PaCO2 increase may suggest an improper functioning of respiratory compensation, and the presence of respiratory acidosis may also be detected. Careful consideration should be given to the possibility that PaCO2 increases and respiratory acidosis might contribute to the severity of COVID-19.

**Response:**

As suggested, we have changed the discussion below (Discussion L247);

There are two possible reasons why elevated bicarbonate is associated with poor prognosis. First, elevated bicarbonate levels may be an early predictor of respiratory acidosis. Increased PaCO2 and respiratory acidosis contribute to the severity of COVID-19. The PaCO2 increased on Day 4 in the high bicarbonate group but only one patient met the definition of respiratory acidosis (ph<7.38 and PaCO2>42mmHg). Higher bicarbonate may predict a subsequent rise in PaCO2 and respiratory acidosis. Another reason is that elevated bicarbonate levels may reflect overactivity of the RAA system in patients with worsening COVID-19. Our results showed that PaCO2 levels compensatorily increased without lowering pH in the high bicarbonate concentration group, which may indicate that high bicarbonate concentrations were related to metabolic alkalosis.

**Reviewer comment**

4) **Diuretics are an important cause of metabolic alkalosis and hypokalemia. The history of diuretic use should be described.**

**Response:**

We had described the history of loop diuretic use before admission in Table 1 (Loop diuretics, 7/60 (12%)). Diuretic use after admission was 3 (23%) in the high bicarbonate group, 2 (6.7%) in the normal group, and 3 (18%) in the low bicarbonate group.

We have added this information as a supplementary table in the appendix, including drugs used other than diuretics.

**Supplementary table1: Drug use after admission**

|  | Overall | High  bicarbonate | Normal  bicarbonate | Low  bicarbonate |
| --- | --- | --- | --- | --- |
|  | n=60 | n=13 | n=30 | n=17 |
| Diuretic, n (%) | 8 (13%) | 3 (23%) | 2 (6.7%) | 3 (18%) |
| Sodium bicarbonate, n (%) | 0 (0%) | 0 (0%) | 0 (0%) | 0 (0%) |
| Transfusion, n (%) | 0 (0%) | 0 (0%) | 0 (0%) | 0 (0%) |
| Steroid, n (%) |  |  |  |  |
| None, n (%) | 45 (75%) | 10 (77%) | 23 (77%) | 12 (71%) |
| Dexamethasone, n (%) | 12 (20%) | 2 (15%) | 6 (20%) | 4 (24%) |
| Prednisolone, n (%) | 3 (5.0%) | 1 (7.7%) | 1 (3.3%) | 1 (5.9%) |
| Remdesivir, n (%) | 0 (0%) | 0 (0%) | 0 (0%) | 0 (0%) |
| Favipiravir, n (%) | 0 (0%) | 0 (0%) | 0 (0%) | 0 (0%) |

Diuretic use was defined as any use of diuretics (loop diuretics, thiazides, K-retaining/anti-aldosterone agents, vasopressin receptor antagonists) at any one time during hospitalization

Also, we have revised results as below;

L176: The history of loop diuretic use before admission was 12%.

L182: Diuretic use after admission was 3 (23%) in the high bicarbonate group, 2 (6.7%) in the normal group, and 3 (18%) in the low bicarbonate group (supplementary table1).

**References**

1.	Kraut JA, Madias NE. Re-Evaluation of the Normal Range of Serum Total CO2 Concentration. Clinical Journal of the American Society of Nephrology. 2018;13(2):343-7.

2.	Theodore AC. Arterial blood gases. In: TW P, editor. UpToDate. UpToDate, Waltham, MA. (Accessed on March 29, 2022.)2022.

3.	Michael Emmett BFP. Simple and mixed acid-base disorders. In: Post T, editor. UpToDate. UpToDate, Waltham, MA, (Accessed on March 29, 2022).

4.	Longenecker JC, Nelson TR. High-yield Acid-base: Lippincott Williams & Wilkins; 2007.

5.	Adrogué H, Madias N. Acid-Base Disorders and Their Treatment. 2005.

6.	Pagana KD, Pagana TJ, Pagana TN. Mosby's® Diagnostic and Laboratory Test Reference: Elsevier - Health Sciences Division; 2020.

7.	Van Smeden M, Moons KG, De Groot JA, Collins GS, Altman DG, Eijkemans MJ, et al. Sample size for binary logistic prediction models: Beyond events per variable criteria. Statistical Methods in Medical Research. 2019;28(8):2455-74.

8.	Pavlou M, Ambler G, Seaman S, De Iorio M, Omar RZ. Review and evaluation of penalised regression methods for risk prediction in low‐dimensional data with few events. Statistics in medicine. 2016;35(7):1159-77.

9.	Puhr R, Heinze G, Nold M, Lusa L, Geroldinger A. Firth's logistic regression with rare events: accurate effect estimates and predictions? Statistics in medicine. 2017;36(14):2302-17.

10.	Mcpherson K, Carlos Iii WG, Emmett TW, Slaven JE, Torke AM. Limitation of Life-Sustaining Care in the Critically Ill: A Systematic Review of the Literature. Journal of Hospital Medicine. 2019.

11.	Vincent J-L, Sakr Y, Singer M, Martin-Loeches I, Machado FR, Marshall JC, et al. Prevalence and Outcomes of Infection Among Patients in Intensive Care Units in 2017. JAMA. 2020;323(15):1478.

12.	Courouble P, Geukens P, Laarbaui F, Beauloye C, Van Caenegem O, Jacquet LM. Adult respiratory distress syndrome caused by 2009 H1N1 influenza during pregnancy: success of ECMO for both the mother and the child. J Extra Corpor Technol. 2011;43(2):75-8.

13.	Yassin Z, Saadat M, Abtahi H, Foroushani AR, Peiman S. Prognostic value of on admission arterial PCO2 in hospitalized patients with community-acquired pneumonia. Journal of Thoracic Disease. 2016;8(10):2765-71.

14.	Elisaf M, Theodorou J, Pappas H, Siamopoulos KC. Acid-base and electrolyte abnormalities in febrile patients with bacteraemia. Eur J Med. 1993;2(7):404-7.

15.	Berend K, De Vries APJ, Gans ROB. Physiological Approach to Assessment of Acid–Base Disturbances. New England Journal of Medicine. 2014;371(15):1434-45.
