# Supplementary material for: Bicarbonate concentration as a predictor of prognosis in moderately severe COVID-19 patients: A multicenter retrospective study
Source: PLoS One. 2022 Jun 24;17(6):e0270141. doi: 10.1371/journal.pone.0270141 (PMC9232226; doi:10.1371/journal.pone.0270141)
Supplement: Attachment — Submitted filename: 2nf Response to Reviewers_20220602.docx [file pone.0270141.s002.docx]

**Reviewer #1:**

**Reviewer comment**

The authors replied to almost all the concerns I pointed out. Nonetheless, I have still concerned about too many fittings in the multivariable Cox regression model.

Generally, events per variable (EPV)>10 is recommended, and even one of the authors' suggested references evaluated EPV=3 (least) and EPV=5 (Stat Med. 2016 Mar 30;35(7):1159-77.) However, the EPV in the multivariable Cox regression model in this study was less than 2, which was extremely low.

In my opinion, the result shown in this study is plausible. However, I do not understand that the authors stuck to the multivariable model. I think univariable or only a few parameters fitting Cox regression models are enough to show the result.

**Response**

Thank you for the comment. We agree with your suggestion that univariable or only a few parameters fitting Cox regression models are enough to show the result. We revised our primary analysis to a model adjusted for only treatment limitations.

Since it would be an outcome reporting bias not to include the results of pre-planned analyses, we would like to remain the results of these sensitivity analyses.

The main results and sensitivity analysis tables and the corresponding text sections have been changed accordingly.

The main results and sensitivity analysis tables were revised as follows;

| **Table 2: The association between bicarbonate concentration and outcomes** | | | | | |
| --- | --- | --- | --- | --- | --- |
|  | Incidence | Crude HR (95% CI) | *p*-value | Adjusted* HR (95% CI) | *p*-value |
| **Primary outcome** |  |  |  |  |  |
| Clinical worsening within 90 days, n (%) † | | | | | |
| High | 7/13 (54%) | 2.98 (1.04 to 8.53) | 0.042 | 3.02 (1.06 to 8.64) | 0.04 |
| Normal | 7/30 (23%) | ref |  | ref |  |
| Low | 11/17 (65%) | 3.80 (1.46 to 9.89) | 0.006 | 3.49 (1.33 to 9.12) | 0.01 |
| **Secondary outcomes** |  |  |  |  |  |
| Intubation within 90 days, n (%) | | | | | |
| High | 6/13 (46%) | 6.10 (1.52 to 24.4) | 0.011 | 6.25 (1.56 to 25.0) | 0.01 |
| Normal | 3/30 (23%) | ref |  | ref |  |
| Low | 6/17 (35%) | 4.47 (1.11 to 18.0) | 0.035 | 4.91 (1.22 to 19.8) | 0.03 |
| Death within 90 days, n (%) | | | | | |
| High | 4/13 (54%) | 2.37 (0.63 to 8.89) | 0.200 | 2.54 (0.68 to 9.49) | 0.165 |
| Normal | 5/30 (17%) | ref |  | ref |  |
| Low | 8/17 (47%) | 4.01 (1.29 to 12.4) | 0.016 | 3.38 (1.08 to 10.5) | 0.035 |
| Clinical worsening within 28 days, n (%) | | | | | |
| High | 7/13 (54%) | 3.09 (1.08 to 8.84) | 0.035 | 3.09 (1.08 to 8.81) | 0.035 |
| Normal | 7/30 (23%) | ref |  | ref |  |
| Low | 10/17 (59%) | 3.54 (1.34 to 9.38) | 0.011 | 3.29 (1.24 to 8.74) | 0.017 |
| HR, hazard ratio; CI, confidence interval;  *Adjusted for treatment limitation.  †Clinical worsening: intubation or death for 90 days. | | | | | |

|  | High bicarbonate | | Low bicarbonate | |
| --- | --- | --- | --- | --- |
| Models | Adjusted HR (95% CI) | *p-*value | Adjusted HR (95% CI) | *p-*value |
| Primary model | 3.02 (1.05 to 8.63) | 0.03 | 3.48 (1.33 to 9.12) | 0.01 |
| Model 1 | 3.27 (1.04 to 10.2) | 0.041 | 4.02 (1.49 to 10.8) | 0.006 |
| Model 2 | 3.24 (1.03 to 10.1) | 0.043 | 3.67 (1.33 to 10.1) | 0.012 |
| Model 3 | 3.22 (1.03 to 10.1) | 0.044 | 3.38 (1.26 to 9.05) | 0.016 |
| Model 4 | 3.29 (1.01 to 10.7) | 0.048 | 4.07 (1.49 to 11.1) | 0.006 |
| HR, hazard ratio; CI, confidence interval, Reference is normal bicarbonate group  The primary model adjusted for treatment limitation,  Model 1 for age, sex, treatment limitation, and respiratory sub- sequential organ failure assessment (SOFA) score;  Model 2 for age, sex, treatment limitation, respiratory sub-SOFA score, and renal sub-SOFA score;  Model 3 for age, sex, treatment limitation, and PaO2/FiO2 ratio;  Model 4 for age, sex, treatment limitation, respiratory sub-SOFA score, chronic pulmonary disease, hypertension, diabetes, and body mass index. | | | | |

**Reviewer #2:**

First, I apologize for the confusion due to my previous comments on Figure 2 and Table 2.

I would like to thank the authors for responding to my initial comments.

I do not have further questions.

**Response**

Thank you.
